# Effectiveness of Workplace-Based Tobacco Awareness Interventions on Quit Readiness Among Bus Drivers: A Randomized Comparative Pilot Trial

**DOI:** 10.7759/cureus.106933

**Published:** 2026-04-13

**Authors:** Gagan Raj, Ankita Jain

**Affiliations:** 1 Department of Public Health Dentistry, Teerthanker Mahaveer Dental College and Research Centre, Teerthanker Mahaveer University, Moradabad, IND

**Keywords:** basic health education, cognitive behavioral therapy, quit readiness, tobacco awareness program, transtheoretical model

## Abstract

Introduction

Tobacco use significantly contributes to the occurrence of morbidity and mortality. Therefore, preventing tobacco use is very important to reduce these health risks. Groups that have jobs (such as bus drivers) have increased susceptibility to tobacco use based on extended work hours, workplace-related stress, sedentary job activities, and social peer pressure, all of which can increase the likelihood of tobacco use. Tobacco education programs that are located at the workplace provide a good opportunity for educating workers about smoking cessation and increasing their knowledge, motivation, and readiness to stop using tobacco.

Aim

This study aims to determine whether cognitive behavioral therapy (CBT)-based tobacco education programs or basic health education (BHE) tobacco education programs are effective for increasing quit readiness among bus drivers.

Methods

A randomized comparative pilot trial compared CBT and BHE interventions on quit readiness among 40 active male smoker bus drivers. Participants were randomized into a CBT-based tobacco awareness program (n = 20) or a BHE-based program (n = 20). The transtheoretical model (TTM) stages of change were assessed at baseline, four weeks, and six months using the validated stages of change questionnaire.

Results

Baseline stage distributions were comparable (p = 0.751). At four weeks, the CBT group showed significantly greater progression toward the preparation and action stages (p = 0.033). At six months, the CBT group demonstrated substantially greater advancement toward the action and maintenance stages (p = 0.001).

Conclusion

Workplace-based tobacco awareness programs incorporating CBT were more effective than BHE in enhancing quit readiness, as measured by TTM stage progression, among bus drivers. These findings should be interpreted with caution, given the pilot design, reliance on self-reported outcomes, and the absence of biochemical verification of cessation. Larger, multicenter trials with longer follow-up and objective cessation measures are needed to confirm these results.

## Introduction

Tobacco use significantly contributes to the occurrence of morbidity and mortality. Therefore, preventing tobacco use is very important to reduce these health risks. It is linked to many different types of systemic illness. This includes heart disease, lung disease, gum disease, and many forms of cancer. Tobacco control efforts have led to a growing awareness of tobacco's dangers; however, despite these efforts, tobacco use remains very common in many developing countries [1]. Tobacco use is especially common among males and working-age populations in India. A large percentage of adults in India consume tobacco in some form, either by chewing or smoking it. Thus, tobacco use remains a major problem, and effective cessation interventions are needed [2].

Groups of people who experience stressful work environments and have irregular work schedules tend to use tobacco more frequently. Bus drivers are a good example of a high-risk occupational group. Their work environment consists of long, irregular hours of driving in heavy traffic [3]. They receive little rest and spend much of their time sitting in vehicles. They are under considerable job-related stress. As a result, they may begin using tobacco as a way to reduce fatigue, increase alertness, and alleviate emotional distress experienced during extended driving hours [4]. In addition, drivers may be encouraged to use tobacco by workplace social norms and limited access to health education services that could help them quit using tobacco [5].

Increasingly, workplaces are being recognized as places where health promotion programs, including tobacco cessation programs, can be implemented. Programs that raise awareness about tobacco use at the workplace offer the potential to target high-risk populations directly in their work environment [6]. Awareness and motivational programs may encourage workers to consider quitting tobacco use. Additionally, such programs can provide workers with access to behavioral support services. Research indicates that workplace-based programs incorporating both educational and behavioral elements can lead to positive attitudes toward quitting and reducing tobacco use [7].

Tobacco dependence is a complex phenomenon that includes both physical addiction to nicotine and behavioral patterns influenced by environmental and psychological factors. Therefore, cessation programs must include interventions that address both the physiological and cognitive behavioral aspects of tobacco dependence [8]. The transtheoretical model (TTM), proposed by Prochaska and DiClemente, conceptualizes behavior change as progression through five stages: precontemplation, contemplation, preparation, action, and maintenance. The maintenance stage is formally defined as sustained abstinence for a minimum of six months, a threshold that corresponds to the six-month follow-up used in the present study [9].

Basic health education (BHE) is a commonly employed approach in tobacco control. BHE programs are primarily educational in nature and are intended to educate participants about the harmful consequences of tobacco use and the benefits of quitting. While BHE programs are effective in increasing awareness and motivating individuals to consider quitting, their impact on sustained behavioral change may be limited because BHE programs do not address the psychological triggers and habitual patterns of tobacco use [10].

Cognitive behavioral therapy (CBT) is a structured, psychologically oriented approach to identifying and modifying ​​​​​maladaptive cognitions and behaviors. CBT is useful in tobacco cessation, as it enables individuals to identify and manage smoking-related triggers, dispute irrational beliefs related to tobacco use, and develop effective coping strategies for managing cravings and preventing relapse [11]. Studies have shown that behavioral counseling approaches that incorporate CBT-based principles significantly improve smoking cessation outcomes over those that involve only minimal intervention or brief advice [12].

Additionally, behavioral counseling has been recommended as an essential component of tobacco cessation strategies by global public health authorities. The World Health Organization emphasizes the importance of providing behavioral support and counseling as part of tobacco control programs to improve the success of quitting attempts [13]. Similarly​​​​​, the US Preventive Services Task Force recommends behavioral counseling as an effective means to assist adults in quitting tobacco use [14].

Research has demonstrated that workplace-based smoking cessation programs can increase employees’ readiness to quit and improve abstinence rates. Programs that combine educational and behavioral counseling components have been shown to produce greater improvements in cessation outcomes than those based solely on information [15]. However, evidence on such programs among occupational drivers in India remains limited.

Government bus depot employees are an important sector of the labor force. They are responsible for public transportation; however, their occupational health needs are often neglected. Given their increased risk of tobacco use due to their occupation and limited opportunities for health promotion, workplace-based tobacco awareness programs may provide a key strategy for promoting cessation behavior. Both educational and behavioral strategies in these programs may be especially helpful in promoting quit readiness and producing lasting behavioral change [16].

Thus, the current randomized pilot study aimed to assess the feasibility and effectiveness of workplace-based tobacco awareness programs delivered through CBT and BHE in increasing quit readiness among bus drivers at the Government Bus Stand in Patna, using TTM stage progression as the primary outcome measure.

## Materials and methods

A pilot randomized controlled trial (RCT; two-arm, parallel-group, single-blinded) was conducted at the Patna Government Bus Stand, India, over six months (September 2024 to March 2025). Participants were blinded to group allocation. Outcome assessment was conducted by a research assistant who was not involved in delivering either intervention and remained blinded to group allocation throughout data collection and coding.

Forty male bus drivers who smoked daily participated. Sample size was based on a medium effect-size estimate of 0.5, α = 0.05, and 80% power, requiring ≥34 participants; 40 were enrolled to accommodate potential dropout. Both groups comprised 20 subjects. Inclusion required current daily smoking and willingness to attend intervention and assessment sessions. Exclusion criteria were exclusive use of smokeless tobacco, current enrolment in a tobacco cessation program, severe psychiatric disorder, or physical inability to attend group sessions.

Participants were recruited by purposive sampling at the Patna Government Bus Stand. Following eligibility screening and written informed consent, subjects were randomized 1:1 into the CBT or BHE group using the lottery method. Allocation concealment was maintained by a third-party researcher not involved in recruitment or data collection; group allocation was revealed only at the commencement of the intervention.

Both programs were delivered in weekly group sessions of 45-60 minutes over four consecutive weeks at the workplace. Sessions were conducted from a prespecified structured manual developed before the study and monitored for content adherence by a senior investigator (A.J.) through review of session notes. Formal fidelity assessment using a validated checklist was not conducted, which is acknowledged as a limitation.

The CBT sessions were facilitated by a qualified public health dentist with formal training in motivational interviewing and cognitive behavioral counseling for tobacco cessation. Sessions were structured according to the TTM and addressed cognitive and behavioral components of tobacco use, including identification of smoking triggers, restructuring of maladaptive beliefs, coping strategy development, goal setting, self-monitoring, and relapse prevention, with emphasis on self-efficacy and behavioral control.

The BHE sessions were delivered by a trained public health educator. These sessions provided information on the adverse health effects of tobacco use, the benefits of quitting, motivational messages, and general cessation guidance, without structured cognitive or behavioral restructuring components.

Quit readiness, operationalized as the TTM stage of change, was the primary outcome. Stage classification was performed using the validated stages of change (SOC) questionnaire. This instrument classifies participants through a validated decision-tree algorithm based on four key questions assessing: (1) current smoking status, (2) intention to quit within 30 days, (3) intention to quit within six months, and (4) prior quit attempts of ≥24 hours.

In accordance with the TTM, participants were grouped into five SOC. The first is the precontemplation stage, which comprises participants who do not intend to quit smoking within the next six months. Nonetheless, defining the precontemplation stage as comprising participants who are willing to quit smoking in six months is inconsistent with the standard TTM definition. The second is the contemplation stage, which comprises participants who are contemplating quitting smoking in the next six months. The third is the preparation stage, which comprises participants who are planning to quit in the next 30 days, having tried quitting before. The fourth is the action stage, which comprises participants who are currently trying to quit smoking or those who have successfully stopped smoking but have done so for less than six months. The fifth is the maintenance stage, which comprises participants who have maintained abstinence from smoking for more than six months. It is pertinent to highlight that at least six months must have elapsed since participants stopped smoking for the TTM to categorize them as being at the maintenance stage.

The TTM stages of behavior change were assessed using a structured tool designed to categorize participants based on their readiness to quit smoking. The assessment was conducted ​​​​​at three key time points: baseline, four weeks, and 12 weeks. The participants' progression through the SOCs - precontemplation, contemplation, preparation, action, and maintenance was evaluated using self-report questionnaires.

Each subject participated voluntarily. Written informed consent was secured from each participant before participation. All personal information of the participants remained confidential throughout the study. Participants were notified that they could terminate their participation at any time without penalty. The Institutional Ethics Committee (IEC) of Teerthanker Mahaveer Dental College and Research Centre, Moradabad, India, approved this study under IEC proposal #003/24.

Microsoft Excel (Microsoft Corp., Redmond, WA) was utilized to enter all data. Data were analyzed using IBM SPSS version 26 (IBM Corp., Armonk, NY). Descriptive statistics were used to provide summaries of stage distribution at each measurement point. Chi-square tests were utilized to compare stage progression between the CBT tobacco awareness program group and the BHE tobacco awareness program group at baseline, four weeks, and 12 weeks. A p-value of less than 0.05 indicated a statistically significant difference.

## Results

A total of 40 male bus drivers participated in the study, with 20 participants allocated to the CBT group and 20 to the BHE group. The mean age of participants in the CBT group was 38.6 ± 7.4 years, while that in the BHE group was 39.2 ± 6.9 years, with no statistically significant difference between the groups (p > 0.05). The majority of participants in both groups had a smoking duration of more than 10 years (CBT: 65%; BHE: 60%). The average number of cigarettes smoked per day was comparable between the groups (CBT: 12.3 ± 3.1; BHE: 11.8 ± 2.9). In terms of educational status, most participants had completed secondary education (CBT: 55%; BHE: 50%), followed by primary education. Baseline characteristics, including age, smoking duration, and daily tobacco consumption, were comparable between the two groups, indicating homogeneity at baseline.

Participants were categorized into one of the five TTM stages (precontemplation, contemplation, preparation, action, and maintenance) at each follow-up (four weeks and 24 weeks) based on their responses to the TTM-based questionnaire. At baseline, participants were categorized according to their initial readiness to quit smoking.

At four weeks and 24 weeks, participants’ progression through the stages was assessed by the follow-up questionnaire. If participants reported increased readiness to quit (e.g., moving from precontemplation to contemplation stage or from preparation to action stage), they were classified accordingly. The categorization allowed for tracking the changes in behavior and readiness over time.

At four weeks, significant shifts were noted in the CBT group, with more participants advancing toward preparation and action stages compared to the BHE group. Similarly, by 24 weeks, a greater proportion of the CBT group progressed into the maintenance stage, indicating a successful transition through the SOCs.

The sequence generation was performed by assigning each participant a unique identification number. A random number generator was used to allocate participants to either the CBT or BHE group. The process ensured that participants were assigned to groups without bias, with a 1:1 allocation ratio between the two groups.

Allocation concealment was ensured by conducting the randomization prior to the intervention, with a third-party researcher handling the process. This researcher was not involved in recruitment or data collection, minimizing the risk of bias. Group allocation was concealed until the start of the intervention, ensuring that the allocation process was not influenced by the researchers or participants.

Stage-wise comparison of behavioral change between CBT and BHE groups

The distribution of participants across stages of behavior change from baseline, four weeks, and 24 weeks is depicted in Table 1. Most participants in both the CBT and BHE groups were in the precontemplation and contemplation stages at baseline. In the CBT group, 60% of participants were in the precontemplation stage and 40% in the contemplation stage, while 55% and 45%, respectively, were in these stages in the BHE group. At baseline, there were no participants in either group in the preparation, action, or maintenance stages. Although the two distributions were similar at baseline (χ² = 0.10, p = 0.751), they indicate that both groups had a similar stage distribution prior to the initiation of the intervention.

**Table 1:** Comparison of stages of change (transtheoretical model) at baseline and follow-up between CBT and BHE groups CBT: Cognitive behavioral therapy; BHE: Basic health education.

Follow-up	Stages of Change	CBT Group (N = 20) n (%)	BHE Group (N = 20) n (%)	Chi-square	P-value
Baseline	Precontemplation	12 (60%)	11 (55%)	0.10	0.751 (NS)
	Contemplation	8 (40%)	9 (45%)		
	Preparation	0	0		
	Action	0	0		
	Maintenance	0	0		
4 weeks	Precontemplation	0	1 (5%)	6.82	0.033 (S)
	Contemplation	3 (15%)	8 (40%)		
	Preparation	9 (45%)	7 (35%)		
	Action	8 (40%)	4 (20%)		
	Maintenance	0	0		
6 months	Precontemplation	0	0	14.95	0.001 (S)
	Contemplation	0	3 (15%)		
	Preparation	2 (10%)	7 (35%)		
	Action	7 (35%)	8 (40%)		
	Maintenance	11 (55%)	2 (10%)		

At four weeks post-intervention, both groups showed a pronounced shift toward later stages of behavior change. However, this shift was more pronounced in the CBT group than in the BHE group. At four weeks, 100% of the CBT group were beyond the precontemplation stage, 15% were still in the contemplation stage, 45% had moved to the preparation stage, and 40% moved to the action stage. In the BHE group, 95% had moved beyond the precontemplation stage, 40% were in the contemplation stage, 35% were in the preparation stage, and 20% had moved to the action stage. This suggests a statistically significant difference in stage distribution between the CBT and BHE groups (χ² = 6.82, p = 0.033).

At six months, the CBT group demonstrated markedly greater progression. No participants in the CBT group remained in the precontemplation or contemplation stages; 10% were in the preparation stage, 35% in the action stage, and 55% had reached the maintenance stage. In the BHE group, 15% remained in the contemplation stage, 35% in the preparation stage, 40% in the action stage, and only 10% reached the maintenance stage. The intergroup difference was highly significant (χ² = 14.95, p = 0.001).

## Discussion

This randomized pilot study found that CBT-based workplace tobacco awareness programs produced significantly greater improvements in quit readiness, as measured by TTM stage progression, compared with BHE among bus drivers. At both follow-up time points (four weeks and six months), participants in the CBT group advanced to later TTM stages at substantially higher rates than those in the BHE group. These findings should be interpreted in terms of enhanced readiness to quit and behavioral stage advancement rather than verified smoking cessation, given the study's reliance on self-report and the absence of biochemical outcome verification.

The behavioral stage-based conceptualization of the TTM, developed by James O. Prochaska and Carlo C. DiClemente, suggests that tobacco cessation is a dynamic process characterized by a series of transitional shifts in the level of preparation toward quitting rather than a sudden and complete behavioral change [17]. Therefore, the present findings support this TTM, as CBT appeared to systematically facilitate stage-based behavioral progression.

Previous research has consistently shown that CBT produces greater levels of smoking cessation success than other types of interventions. For example, a Cochrane collaboration meta-analysis of behavioral counseling versus minimal intervention or brief advice conducted by Lancaster and Stead found that behavioral counseling resulted in substantially greater smoking cessation success than either of these less intensive forms of assistance [18]. Furthermore, Robert West has argued that structured behavioral interventions increase motivation, coping mechanisms, and relapse prevention skills in smokers and therefore result in increased quit success [19].

The structured nature of CBT was most likely responsible for the outcomes observed in the present study. By identifying and modifying maladaptive cognitive processes related to smoking, by reducing stress-related triggers for smoking, and by enhancing adaptive coping skills, CBT addresses both the psychological dependence on cigarettes and the conditioned behavioral responses associated with cigarette consumption [20]. In contrast, BHE primarily enhances knowledge and awareness about smoking and its consequences and does not provide adequate support for changing established, long-standing behavioral reinforcement patterns that maintain smoking [21].

The WHO and United States Preventive Services Task Force (USPSTF) have both endorsed behavioral counseling, including CBT-based approaches, as essential components of tobacco cessation programs [22]. The present findings among occupational drivers support this recommendation. The high proportion of CBT participants who reached the maintenance stage (55%) at six months indicates strong potential for structured behavioral counseling within workplace health promotion programs.

Drivers are at particularly high risk for developing tobacco use disorders due to occupational stress, long work hours, disrupted sleep patterns, and peer pressure. Previous occupational health research has demonstrated that providing workplace-based behavioral interventions can lead to improved readiness to quit and abstinence rates [23]. The large number of drivers who were able to advance to the maintenance stage (55%) in the CBT group at the 24-week time point indicates that there is strong potential for using structured behavioral counseling as part of workplace-based health promotion programs.

Numerous trials have demonstrated that CBT-based interventions produce sustained smoking cessation by achieving higher quit rates and lower relapse rates than minimal intervention or brief advice [24,25]. The relapse-prevention components of CBT have also been shown to produce sustained benefits for cessation maintenance.

In general, the findings of the present study reinforce the existing body of global evidence supporting the idea that CBT-based interventions are more successful than information-only approaches in achieving sustained changes in behaviors among tobacco users.

Limitations

This study has several limitations. First, as a pilot study, the sample size was very limited, which** **reduces the power of the study and limits the ability to detect meaningful differences. Second, the study was conducted at a single work site; therefore, the findings cannot be generalized across other work sites or occupational groups. Third, purposive sampling was used to select participants, which introduces the possibility of selection bias, as participants may not represent the broader population of bus drivers or smokers. This affects the generalizability of the results. Fourth, quit readiness was assessed using self-report questionnaires. Self-reported stage classification is susceptible to social desirability bias and potential misclassification of participants' true stage of behavioral change. Fifth, the study did not include biochemical verification of smoking cessation (e.g., carbon monoxide testing or cotinine measurement). Without objective verification, the accuracy of self-reported cessation status cannot be confirmed. Sixth, the study did not employ a formal, validated intervention fidelity assessment tool. Although sessions were delivered using a prespecified structured manual and monitored for content adherence by a senior investigator, the absence of a validated fidelity checklist means the degree of protocol adherence cannot be confirmed. Future studies should incorporate formal fidelity monitoring to strengthen internal validity. Finally, future research should include large-scale, multicenter randomized controlled trials with longer follow-up, biochemical verification of cessation, and formal fidelity assessment to provide definitive evidence of program effectiveness and long-term relapse prevention.

## Conclusions

This pilot randomized trial provides preliminary evidence that workplace-based CBT tobacco awareness programs are more effective than BHE in improving quit readiness, as measured by TTM stage progression, among bus drivers. Participants in the CBT group demonstrated substantially greater advancement from the precontemplation and contemplation stages to the preparation, action, and maintenance stages over six months compared with those receiving BHE.
